# Finding the *Unicorn*, a New Mouse Model of Midfacial Clefting

**DOI:** 10.3390/genes11010083

**Published:** 2020-01-11

**Authors:** Brandi Lantz, Casey White, Xinyun Liu, Yong Wan, George Gabriel, Cecilia W. Y. Lo, Heather L. Szabo-Rogers

**Affiliations:** 1 Center for Craniofacial Regeneration, Department of Oral Biology, School of Dental Medicine, University of Pittsburgh, Pittsburgh, PA 15213, USA; brg77@pitt.edu (B.L.); caw176@pitt.edu (C.W.); xil213@pitt.edu (X.L.); yow11@pitt.edu (Y.W.); 2 College of Dentistry, The Ohio State University, Columbus, OH 43210, USA; 3 Department of Developmental Biology, School of Medicine, University of Pittsburgh, Pittsburgh, PA 15213, USA; gcg9@pitt.edu (G.G.); cel36@pitt.edu (C.W.Y.L.); 4 McGowan Institute for Regenerative Medicine, Pittsburgh, PA 15219, USA

**Keywords:** midfacial clefting, Unicorn, medial nasal prominences, nasal septum, *Raldh2*, *Leo1*

## Abstract

Human midfacial clefting is a rare subset of orofacial clefting and in severe cases, the cleft separates the nostrils splitting the nose into two independent structures. To begin to understand the morphological and genetic causes of midfacial clefting we recovered the *Unicorn* mouse line. *Unicorn* embryos develop a complete midfacial cleft through the lip, and snout closely modelling human midfacial clefting. The *Unicorn* mouse line has ethylnitrosourea (ENU)-induced missense mutations in *Raldh2* and *Leo1*. The mutations segregate with the cleft face phenotype. Importantly, the nasal cartilages and surrounding bones are patterned and develop normal morphology, except for the lateral displacement because of the cleft. We conclude that the midfacial cleft arises from the failure of the medial convergence of the paired medial nasal prominences between E10.5 to E11.5 rather than defective cell proliferation and death. Our work uncovers a novel mouse model and mechanism for the etiology of midfacial clefting.

## 1. Introduction

Orofacial clefting (OFC) is the most common congenital craniofacial anomaly present at birth. OFC is a group of anomalies that has many subtypes with much phenotypic variation. The most frequently observed anomaly is the lateral cleft lip and palate, which occurs in approximately 1/700 live births [1]. By comparison, midfacial clefting (Tessier type 0) are exceptionally rare, with an estimated prevalence of 5/100,000 live births [2]. In humans, the spectrum of midfacial clefting phenotypes can range from a mild phenotype with isolated midline notched upper lip to the severe phenotype with a midline cleft extending from the upper lip, through the philtrum and nasal septum resulting in independent nostrils and hypertelorism [2]. The milder phenotypic forms are likely not reported because they do not require surgical intervention resulting in an underestimation of the true prevalence of midfacial clefting. Still, the disparity in prevalence rates between lateral facial clefting relative to midline clefting has resulted in considerably more research attention being focused on the former. Accordingly, while much light has been shed on the genetic and molecular etiology of lateral cleft lip and palate (reviewed in [3,4]), far less is known about the mechanisms underlying midline clefting.

Embryonic facial development is a highly coordinated process involving paired facial prominences that fuse into a distinct structures through epithelial to mesenchymal interactions [5]. The structures in the adult face that are disrupted in midfacial clefts (the nose, philtrum, midline of the upper lip, and the primary palate) develop from the paired medial nasal prominences (MNP) in the embryo. The process of MNP merging is completed by the seventh week of human gestation, or by embryonic day 11.5 (E11.5) in the mouse. The location of the cleft and the morphology of the skeletal elements that are affected in midfacial clefting support the hypothesis that medial convergence of MNP is necessary for normal midfacial development. However, the genetic and morphological processes that contribute to the development of the midline of the face is not well studied.

At embryonic day (E)10.5 in mice, the mesenchyme within the maxillary prominences begins to rapidly proliferate, thus pushing the MNPs toward the midline of the face [4]. The MNPs are composed of an inner core of mesenchyme that is surrounded by an outer epithelial layer. At least two epithelial signaling centers provide patterning information to the MNPs-the nasal pits, and the frontonasal ectodermal zone (FEZ) [6,7,8]. The most posterior region of MNPs is made of the FEZ, a region of ectoderm that expresses *Sonic Hedgehog* (*Shh)* and *Fibroblast growth factor–8 (Fgf8)*, and determines the direction, orientation, and width of the midline of the face [6,7]. The nasal pits secrete signaling information to induce the surrounding bones and cartilages [8]. In addition to HH signaling in the FEZ, increased hedgehog (HH) signaling is associated with hypertelorism, and in some cases the development of a midfacial cleft [9,10,11,12,13]. The final step for midfacial convergence is the disappearance of the epithelial seam surrounding the MNPs permitting mesenchymal merging [4].

In addition to the morphogens in the FEZ, several other morphogens such as retinoic acid (RA) are required for normal craniofacial morphogenesis. RA is a diffusible molecule that binds to nuclear receptors in the cytoplasm and transits into the nucleus. In the nucleus the RA in complex with co-receptors binds to RA-response elements (RARE) in the promoters of target genes such as *Fgf8* and facilitate transcription (reviewed in [14]). RA is synthesized by members of the retinaldehyde dehydrogenase (RALDH) family and degraded by the Cyp26 family [14]. RA is teratogenic in either high or low doses [14,15,16]. *RALDH2^-/-^* mouse mutants develop frontonasal truncation and major heart defects, but do not survive for the evaluation of bone and cartilage development [17].

Normal facial morphogenesis also relies on the global control of transcription and nuclear organization. Polymerase-associated factor 1 complex (Paf1C) is associated with RNA polymerase II (RNAPII) and functions to improve transcriptional efficiency by promoting the chromatin remodeling [18,19]. The PAF1 complex consists of five proteins *Leo1*, *Rtf1*, *Cdc73*, *Ctr9,* and *Paf1*. Intriguingly, the loss of function of either *rtf1* and *ctr9* members in Zebrafish results in a defect in neural crest, cardiac, and somite development [20]. Zebrafish *leo1* loss of function mutants have decreased chondrogenesis and a midline cleft in the ethmoid plate [21].

Midfacial clefting has not been well characterized clinically in humans postnatally, nor mechanistically within animal models prenatally [2]. Inadequate characterization of this broad-spectrum phenotype could be in part due to a lack of understanding of early nasal septum morphogenesis and MNP merging. Here, we present the *Unicorn* mouse line as a model of human midfacial clefting. Using the *Unicorn* mutants, we determined the critical period for midfacial morphogenesis, and how the placement of the frontonasal ectodermal zone determines the angle of nostril outgrowth. The *Unicorn* mouse line will help to facilitate further understanding of this rare anomaly and thus may contribute to the eventual development of novel treatments.

## 2. Methods

### 2.1. Unicorn Mouse Line Recovery and Husbandry

In brief, the *Unicorn* mouse line was recovered through an ethylnitrosourea (ENU)- mutagenesis screen for late embryonic cardiovascular phenotypes [22,23]. The original phenotyping, exome sequencing, and cryopreservation were performed as described [23]. We recovered founder *Unicorn* (b2b1941Clo) animals at Jackson Laboratory and maintained the line on an outbred C57/BL6J background. We designed our breeding schema to first test the heritability of the midfacial cleft phenotype and to dilute the neutral ENU-induced mutations. Every generation we performed a sibling intercross to ensure heritability of the midfacial cleft and to select for a productive sire. The productive sires were bred with wildtype C57/BL6J females for the next generation. The original ENU treatment was designed to induce 100 mutations [23], and we performed eight generations of outcrossing to select for 1 or 2 causative mutations. We used the exome sequencing of the first generation of phenotypic animals to design custom Invitrogen SNP assays for genotyping with the TaqMan Genotyping MasterMix and read the results on a StepONE touch machine. 

### 2.2. Embryo Collection

We performed timed matings and designated the presence of a plug as embryonic day 0.5 (E0.5). The pregnant dams were euthanized via CO_2_ asphyxiation on the appropriate day and embryos were harvested via C-section. Embryo staging was confirmed by morphology. Animal care and use was conducted in accordance to the animal protocols approved by the Institutional Animal Care and Use Committee of the University of Pittsburgh (protocol 17050839). Embryos were collected and fixed overnight in 4% PFA. 

### 2.3. Skeletal Preparations

Heads of e15.5 *Unicorn* and littermate control embryos were fixed in 95% ethanol and stained with alizarin red and alcian blue using standard protocols [24]. Samples were stored and imaged in a glycerol/ethanol solution.

### 2.4. Pseudo-SEM and Morphometric Analysis

E10.5 and E11.5 *Unicorn* and littermate control embryos were processed and imaged via pseudo-SEM protocol (*n* = 3/genotype, E10.5; *n* = 4/genotype, E11.5). The embryos were fixed overnight in 4% paraformaldehyde, and stained with 0.01% ethidium bromide. To ensure consistency of embryo position for photography, we photographed them in a Sylgard-coated dish with molded wells for embryo placement. We visualized the ethidium bromide signal using the DsRED filter on a Leica MZFLIII. After photography, we placed twelve landmarks on the images, and used the measure tool in Image J version 1.51w to measure the width of the face. We measured the width of several inferior and superior regions as well as the width of the singular maxillary prominence. We normalized each facial width measurement to the singular maxillary prominence width to account for subtle staging differences between individuals. The *Unicorn* and control embryos normalized facial widths were compared using a paired Students *t*-test (*p* < 0.05).

### 2.5. Histology Analysis

For histological analysis, E10.5, E11.5, E15.5 embryos were collected, fixed in 4% PFA overnight, dehydrated and stored in 70% ethanol, and then embedded in paraffin wax [25]. Embryos were sectioned frontally at 10 µm and placed on triethoxysilylpropylamine (TESPA)-coated Super-Frost coated slides. Adjacent slides were stained with hematoxylin and eosin, or Picrosirius red and alcian blue using standard protocols.

### 2.6. In Situ Hybridization

Section in situ hybridization was performed using standard protocols with digoxigenin-labelled RNA in situ probes. After washing to reduce non-specific antibody binding, the location of in situ probes were detected with BM Purple [26]. The following in situ probes were used: *Alkaline phosphatase*, *Runx2*, *Sox9*, *Ptc1*, *Gli1*, and *Erm1* [24,25]. At least 3 littermates were analyzed per each RNA probe. The *Leo1* in situ probed was cloned from cDNA using these primers: Leo1 F:GAAGGGTCTGAAAAAGCCCAG and Leo1R:GGACATGCTTCCATCTGACCA. 

### 2.7. Proliferation and Apoptotic Studies

To detect cells in mitosis we performed immunostaining for Phospho Histone H3 (PHH3). We performed citrate buffer antibody retrieval prior to overnight incubation with Phospho Histone H3 (Cell Signaling #9701S) antibody. The primary antibody signal was amplified using the Vectastain amplification kit, developed in ImmPACT DAB, and the slides were mounted in Thermo Scientific mounting medium. 

To detect cells in S-phase, we i.p. injected 10 mg/kg of BrdU into the pregnant dams one hour prior to embryo collection. Enzymatic antigen retrieval was performed with Exonuclease III, Dpn1, and Proteinase K, followed by incubation with the anti-BrdU antibody (RPN202, GE Healthcare Life Sciences, Pittsburgh, PA, USA). We detected the antibody with Alexa Fluor 488 conjugated secondary antibody (Invitrogen, Carlsbad, CA, USA, A11059) and the slides were mounted in Prolong-Gold with Dapi (Invitrogen).

Apoptotic cells were detected using the terminal deoxynucleotidyl transferase dUTP nick end labelling (TUNEL) using the ApopTag Plus Peroxidase In Situ Apoptosis Detection Kit (EMD Millipore, Burlington, MA, USA).

The analysis of proliferation and apoptosis was performed in a cranial and a caudal region in E10.5 and E11.5 in the MNP mesenchyme and medial epithelial regions. The proliferating cell ratio was calculated as the number of BrdU positive cells divided by the total number of cells. TUNEL- and PHH3- positive cells were counted on adjacent sections and the absolute number was plotted in scatterplot. To analyze proliferation and apoptosis, we counted the positive cells from at least 3 littermates per assay and stage. We performed a Chi-squared test to evaluate the differences in PHH3 or TUNEL-positive cells between *Unicorn* and controls (*p* < 0.05). For the BrdU labeling we compared the *Unicorn* and controls littermates with a paired Students *t*-test (*p* < 0.05).

### 2.8. Imaging

Whole mount images were captured on a Leica M165FC dissecting microscope using a DFC 450 camera and Leica LAS software. Histological images were captured on a Zeiss AXIO microscope with an AxioCam MRc 35 camera and Zen software. Fluorescence images were obtained at the Center for Biological Imaging at the University of Pittsburgh on an Olympus FluoView 1000 confocal and deconvolved using NIS software. Confocal images are presented as maximal projection stacks.

## 3. Results

### 3.1. Unicorn Mice Develop a Midfacial Cleft with a Bifurcated Nasal Septum

We selected the *Unicorn* mouse line for cryorecovery because the initial screen produced two phenotypic embryos with a spectrum of orofacial clefting phenotypes including a midfacial cleft and cleft palate. To fully capture the orofacial clefting phenotype and test the heritability of the orofacial clefting phenotype we devised a breeding schema that involved the analysis of E15.5 embryos from sibling intercrosses to test the heritability of the phenotype. We collected these embryos at E15.5, because lip and palate development is complete and any orofacial phenotype would be obvious at this stage.

**Figure 1:**
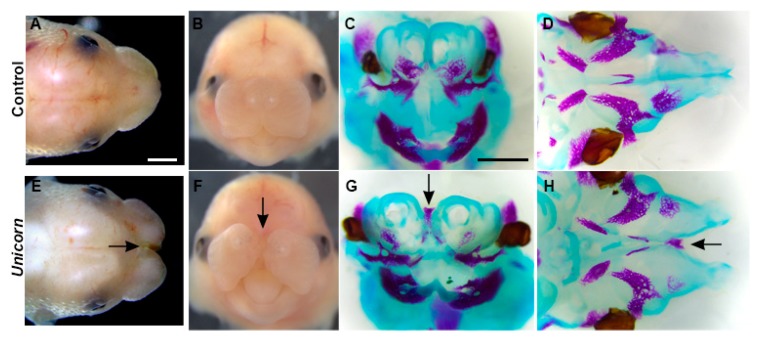
Midfacial cleft phenotype in *Unicorn* mutants. External and bone and cartilage morphology at E15.5 of (**A**–**D**) littermate control and (**E**–**H**) *Unicorn* mutants. (A,E) Dorsal view; (B,C,F,G) frontal view; (D,H) palatal view. (A,B,E,F) External morphology of embryos; the midfacial cleft is indicated by arrow. (C,D,G,H) Skeletal preparations with alizarin red and alcian blue to stain the bone and cartilage respectively. (C,G) At the proximal region of the midfacial cleft, the *Unicorn* mutants have developed bone between the two nasal regions (arrowhead). Nasal conchae and Meckel’s cartilages as well as the premaxillary, maxillary, and mandibular bones are present in *Unicorn* mutants. (D,H) The midline nasal septum is apparent in the control littermate in the palatal view while it is bifid in the *Unicorn* mutant at E15.5 (arrowhead). Scale bars in A = 1 mm, applies to B, E, F. in C = 1 mm and applies to D, G, H.

All of the *Unicorn* mutants collected after the cryorecovery develop a midfacial cleft that extends through the lip, palate, and snout (Figure 1E,F). Except for the midline cleft, the external anatomy of the *Unicorn* mutants is grossly normal, including the nostrils and the lateral sides of the lip (Figure 1F). Additionally, the patterning of the facial bones and cartilages is largely normal. However, because of the midfacial cleft, the premaxilla and vomer of the *Unicorn* mutants are laterally displaced compared to the littermate control at E15.5 (Figure 1G,H). We observed the presence of bone between the bifid nasal septa in *Unicorn* mutants (Figure 1G,H).

### 3.2. Genetics of the Unicorn Mouse Line

In addition to the morphological analysis, the initial two phenotypic embryos were exome sequenced (Supplementary Materials). The exome sequencing uncovered 19 genes that had ENU-induced damaging mutations in common (5 homozygous and 14 heterozygous). We designed our sibling intercross heritability experiment to collect recessive phenotypic embryos at late stages of development (E15.5), and we reasoned that the genes that were present as homozygous mutations in the phenotypic embryos would be causative in our phenotype [23].

We developed custom Invitrogen SNP assays for genotyping five genes (*Cox2*, *Cyp2j13*, *Raldh2*, *Leo1*, and *TMEM106)* and genotyped the first five generations of sibling intercrosses for all genes. By the fifth generation, the midfacial phenotype only segregated with missense mutations in *Raldh2* (c.A1231T:p.M411L) and *Leo1* (Leo1:NM_001039522:exon11:c.T1896A:p.D632E). *Leo1* and *Raldh2* are within 4.2 megabases of each other on mouse Chromosome 9 and we have been unable to separate them in the *Unicorn* line. 

**Figure 2:**
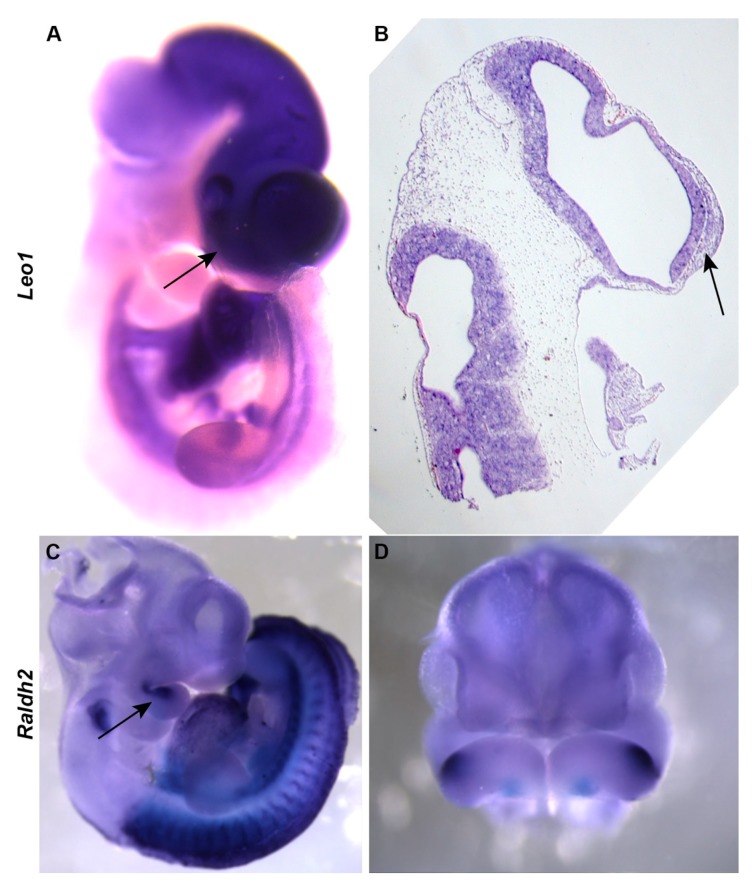
*Leo1* and *Raldh2* are expressed in the facial prominences at E10.5. (**A**,**B**) *Leo1* is expressed strongly in the brain and heart tissue, as well as the medial nasal prominence mesenchyme (arrow). (**C**,**D**) RALDH2 is expressed in the maxillomandibular cleft (arrow) and the brain at E10.5.

Raldh2 synthesizes retinoic acid and is involved in the development of the frontonasal process, secondary palate, and skull vault in mouse embryos [17,27,28]. Leo1 is a subunit of the RNA polymerase II complex, and the IMPC phenotyping reported that homozygotes have a pre-weaning mortality. All phenotypic *Unicorn* mutants are homozygous for both *Leo1* and *Raldh2* ENU-induced mutations. We sought to determine if *Leo1* and *Raldh2* expression patterns overlapped in the developing face. At E10.5. *Raldh2*, and *Leo1* are expressed in and around the converging medial nasal prominences (Figure 2).

### 3.3. Unicorn Nasal Septum and Surrounding Tissues Differentiate Normally

**Figure 3:**
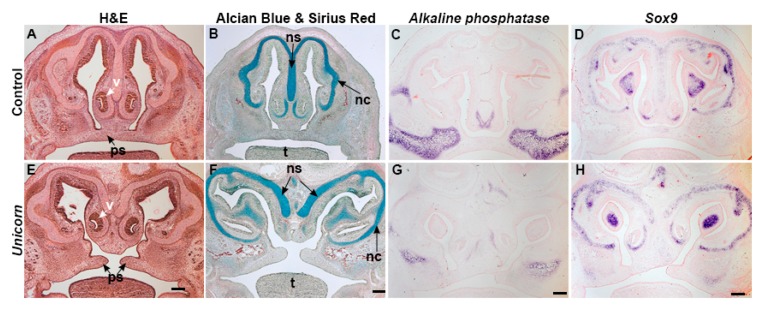
The nasal septum is bifid in the *Unicorn* animals at e15.5. Frontal sections capturing the nose and palate of *Unicorn* and control embryos, stained with (**A**,**E**) hematoxylin and eosin, (**B**,**F**) alcian blue and sirius red, (**C**,**G**) *Alkaline phosphatase* (**D**,**H**) *Sox9*. (A,B,E,F) H&E and alcian blue and sirius red staining reveal the nasal septum as one midline organ located in the frontonasal process in control embryos. In *Unicorn* embryos, there are two individual nasal septa organs located in the frontonasal process. (C,G) *Alkaline phosphatase* expression is present in the maxillary bone, vomer and superior to the nasal conchae of *Unicorn* mutants. (D,H) *Sox9* expression is expressed in the duplicated nasal septum in *Unicorn* mutants. Scale bars = 200 µm. nc, nasal conchae; ns, nasal septum; ps, palatal shelves; t, tongue; v, vomer.

To better understand the morphological cause of the *Unicorn* phenotype, we assayed the histology of bone and cartilage at E15.5. In controls at E15.5, the nasal septum was present as one midline organ that stained strongly with alcian blue, and expressed the chondrocyte marker, *Sox9*. The nasal cartilages were supported by *Alkaline phosphatase* positive bones. The nasal septum and palatal shelves were continuous in controls (Figure 3A–D). The two nasal septa of the *Unicorn* embryos were located within a widened frontonasal process (Figure 3E–H) and were alcian blue positive and expressed *Sox9*, (Figure 3E–H). At E15.5, the *Unicorn* palatal shelves had elevated above the tongue, but had not yet fused with each other or with the nasal septum (Figure 3E–H). In addition, the differentiating bony skeleton surrounding the nasal cartilages expressed *Alkaline phosphatase* and had begun differentiating in relatively normal anatomical positions within the duplicated nostrils. Combined with the skeletal preparations and these analyses we conclude that the *Unicorn* mutant nasal skeleton was developing normally, except that it was shifted laterally.

### 3.4. Unicorn Patterning is Normal at E12.5

**Figure 4:**
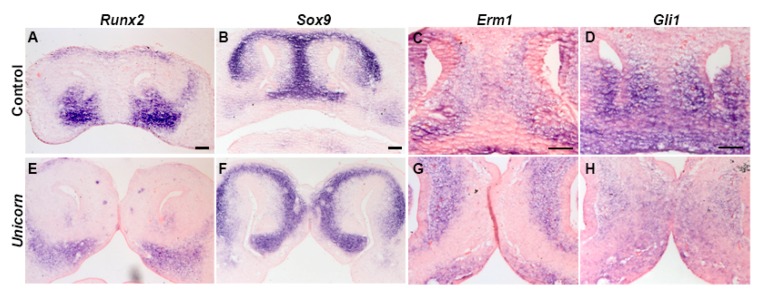
The *Unicorn* nasal mesenchyme has normal expression pattern of markers. (**A**,**E**) *Runx2* is expressed in the premaxilla of *Unicorn* and control embryos. (**B**,**F**) *Sox9* is expressed in the control nasal septum and in both nasal septa of the *Unicorn* embryos. (**C**,**G**) Similar to controls, *Unicorn* embryos express *Erm1* in the mesenchyme adjacent to the *Sox9*-positive region, but is expanded superiorly between the oral ectoderm and cartilaginous condensations. (**D**,**H**) *Gli1* expression surrounds the *Sox9* region in both *Unicorn* and control animals. Scale bars are 100 µm.

Because we observed normal bone and cartilage differentiation at E15.5, we wanted to identify when the bifid nasal septum first arises. During normal development the paired medial nasal prominences coalesce medially by E12.5. The premaxillary bones inferior to the nasal slits in control and *Unicorn* mutant embryos expressed *Runx2*, an osteoblast specific transcription factor, although the *Unicorn* embryos expression domains extended laterally (Figure 4A,E). In control embryos, *Sox9* expression was found in the nasal septum and cartilaginous fins that extend from it (Figure 4B). However, in *Unicorn* mutants, the nasal septum already consisted of two *Sox9*-positive organs (Figure 4F).

We next asked where active Fibroblast growth factor (FGF) signaling and Hedgehog (HH) signaling is occurring at E12.5 with the expression of *Erm1* and *Gli1* expression. We observed that *Erm1* was expressed at similar levels in the mesenchyme surrounding the nasal pit and the nasal septum (Figure 4C,G). The *Erm1* domain was expanded superiorly between the oral ectoderm and cartilaginous condensations of the *Unicorn* embryos (Figure 4G). *Gli1* was expressed in similar mesenchymal domains of *Unicorn* and control E12.5 embryos (Figure 4H). These data suggest that FGF and HH signaling is largely unchanged in the *Unicorn* mutant facial prominences.

### 3.5. The Critical Period for Midfacial Clefting Is between E10.5 and E11.5

**Figure 5:**
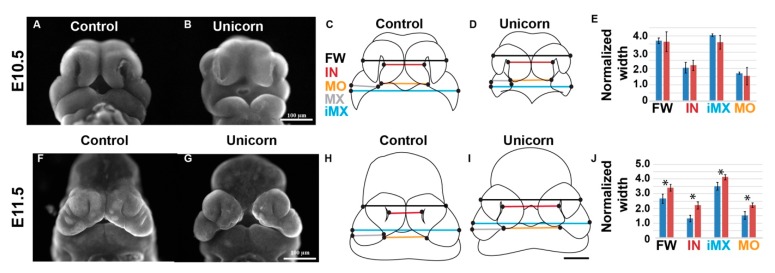
Critical period for midfacial clefting is between E10.5 and E11.5. (**A**,**B**,**F**,**G**) Frontal pseudo-SEM images of E10.5 and E11.5 *Unicorn* and littermate control embryos. At E11.5, the medial nasal prominences (MNPs) in the control mice have met at the midline, while the *Unicorn* mice have a large internasal distance that separates the two MNPs. (**C**,**D**,**H**,**I**) Schematics of E10.5 and E11.5 of the measurements made on control and *Unicorn* faces. We placed 12 landmarks (black circles) on each face and measured the width of face between the lateral edges of the lateral nasal prominences (FW), the width between the medial edges of the nasal pits (IN) the mouth opening width (MO), the width of individual maxillary prominences (MX), and the width between the lateral edges of the paired maxillary prominences (iMX). (**E**,**J**) We normalized the facial widths to the width of one maxillary prominence to account for staging differences between littermates. By E11.5 the normalized internasal distance (IN) is significantly wider in *Unicorn* mutants compared to control. The remainder of the measurements that include the IN are also significantly wider in the *Unicorn* mutants. FW, facial width; IN, internasal distance; MO, mouth opening width; iMX, intermaxillary distance. Scale bars are 100 µm.

At E10.5 mouse MNPs begin to shift from a lateral to a more medial position prior to the medial edges making contact at E11.5. We hypothesized that the midfacial cleft phenotype in the *Unicorn* embryos arose in part because of the MNPs failing to fuse at the midline. To analyze the facial morphology changes occurring at E10.5 and E11.5, we placed facial morphometric landmarks upon the faces of at least three *Unicorn* and control embryos (Figure 5). We measured the facial widths between the paired prominences at different anterior-posterior levels that included the paired MNPs, MXPs, and LNPs. We measured the width of individual maxillary prominences, and found no difference between the width of the maxillary prominences of control and *Unicorn* embryos at E10.5 and E11.5 (E10.5 WT 4.67 µm ± 0.20 µm, *n* = 3; vs *Unicorn* 5.02 µm ± 0.64 µm, *n* = 3; E11.5 WT 7.78 µm ± 1.09 µm, *n* = 4; *Unicorn* 6.96 µm ± 0.95 µm, *n* = 4). Therefore, we normalized the facial widths to a maxillary prominence width to account for staging and size differences in the embryos.

At E10.5, the nasal pits of the *Unicorn* embryos faced laterally, projecting toward the sides of the face, whereas in the littermate control, the MNPs were shifted medially toward the midline of the face (Figure 5A,B). Importantly, the E10.5 *Unicorn* midfacial width is not significantly different from the control embryos (Figure 5E). 

In the E11.5 control embryos, the MNPs had made contact at the midline of the face and are beginning to merge together (Figure 5F). In contrast, in *Unicorn* mutants, MNPs had shifted toward the front of the face, but there was already a significant distance between the medial edges of the MNPs (Figure 5G). We found that the intranasal distance is significantly wider in the *Unicorn* mutants compared to the control and every measurement that includes it is also significantly wider (Figure 5J). These data suggest that the critical period for MNP convergence occurs between E10.5 and E11.5 in the mouse embryo.

### 3.6. Unicorn Embryos Have Decreased Proliferation in Caudal MNPs

**Figure 6:**
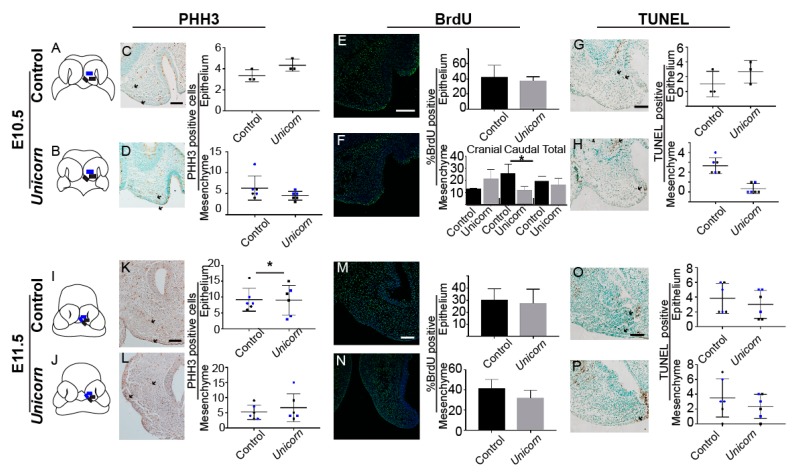
*Unicorn* embryos have decreased caudal mesenchymal and epithelial proliferation at E10.5. Proliferation and apoptosis in the MNP mesenchyme and epithelium at E10.5 (**C**–**H**) and E11.5 (**K**–**P**) during the critical period of MNP fusion. Proliferating cells detected by phospho-Histone H3 (PHH3) and BrdU. TUNEL was used to detect apoptotic cells. (**A**,**B**,**I**,**J**) Schematics of coronal sections showing the regions used for counting the positive cells. The cranial regions are blue, and the caudal regions are black. (C,D) There is no difference between the number of PHH3-positive cells in the E10.5 control or *Unicorn* MNP epithelium and mesenchymal cells. (K,L). There are more PHH3-positive cells in the *Unicorn* MNP epithelium at E11.5 (*p*-value = 0.0274), and no difference in mesenchymal proliferation. (E,F). There are fewer BrdU labeled cells in the caudal portion of the E10.5 MNP mesenchyme, however there is no difference when the two segments are combined nor in the epithelium. (M,N) BrdU incorporation is similar in the epithelium and mesenchyme of *Unicorn* and control MNPs at E11.5. (G,H,O,P) The level of apoptosis in the MNP medial edge epithelium and mesenchyme is similar between control and *Unicorn* MNP at E10.5 and E11.5. Scale bars = 100 µm.

We hypothesized that the difference in morphology that arises between E10.5 and E11.5 could come about through increased proliferation in the *Unicorn* embryo facial prominences. We determined the rate of cellular proliferation using two methods: phospho-Histone H3 immunohistochemistry and the incorporation of Bromodeoxyuridine (BrdU). We chose to use phospho-Histone H3 to detect the cell actively dividing and the BrdU to detect cells that are entering the cell cycle [29,30]. From both E10.5 and E11.5 coronal sections, we counted the number of cells proliferating in a cranial and caudal segment of MNP mesenchyme (Figure 6A,B,I,J). To determine the level of epithelial proliferation, we counted one area at E10.5, and two areas in the MNP medial edge epithelium adjacent to the mesenchymal regions (Figure 6A–D).

At E10.5, *Unicorn* MNP mesenchyme showed significantly less proliferation within the caudal mesenchyme when evaluated via BrdU (Figure 6D,F). By contrast, levels of phospho-Histone staining in the same regions was unchanged. By E11.5, we detected no significant differences in proliferation between the control and *Unicorn* MNP mesenchyme (Figure 6N). There was no difference in the epithelial proliferation rate at E10.5 (Figure 6C,E). We observed significant differences within the caudal epithelium at E11.5 when evaluated via PHH3 (Figure 6I).

In the secondary palate, there is evidence that the medial edge epithelial cells disappear via apoptosis [31,32,33,34]. We used TUNEL (terminal deoxynucleotidyl transferase (TdT) nick end labeling) assay to detect apoptotic cells in the MNPs. We quantified apoptotic cells in the same regions as the proliferating cells and we did not observe any significant differences in the epithelium nor mesenchyme of the *Unicorn* embryos at either timepoint (Figure 6G,H,O,P). We did observe many TUNEL-positive cells located in the lateral lip zone of fusion (Figure 6O,P).

### 3.7. Unicorn Frontonasal Ectodermal Zone Is Shifted Laterally

**Figure 7:**
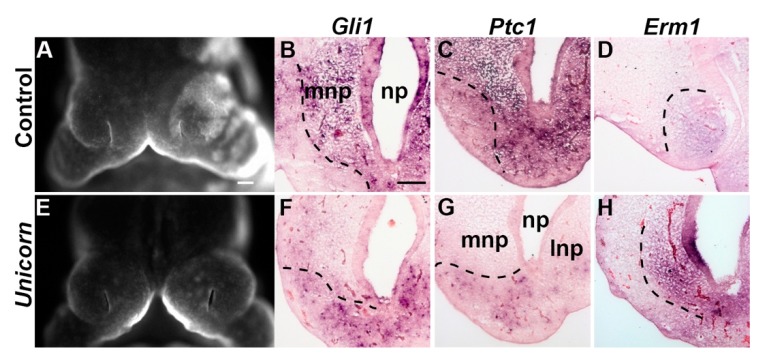
The *Unicorn* FEZ is shifted laterally. Wholemount images of embryos used for section in situ hybridization to HH reporters, *Ptc1*, *Gli1,* and FGF reporter, *Erm1* at E11.5 in control (**A**–**D**) and *Unicorn* (**E**–**H**) littermates. (A,E) Pseudo-SEM images of the face of the embryos used for the in situ analysis. (B,F) *Gli1* expression domain is shifted laterally in *Unicorn* mutants. (C,G) *Ptc1* domain is more lateral and distal to the nasal pit epithelium. (D,H) *Erm1* expression domain is shifted laterally and anteriorly in *Unicorn* mutants. Scale bar in A = 200 µm and applies to E; B = 100 µm and applies to C, D, F–H.

The frontonasal ectodermal zone (FEZ) is a signaling center that determines the orientation and direction of outgrowth of the upper jaw [6,7]. The FEZ is an epithelial signaling center that expresses complimentary domains of *Fgf8* and *Shh*. To test the location of the functional FEZ, we evaluated the expression of HH-responsive genes: *Glioma-associated oncogene homolog 1* (*Gli1*), and *Patched1* (*Ptc1*), and FGF-responsive gene: *Ezrin-radixin-moesin* (*Erm1*) at E11.5. In *Unicorn* mutants the domains of both *Gli1* and *Ptc1* is shifted caudally and laterally compared to the control MNP at E11.5 (Figure 7B,C,F,G). Correspondingly, the expression of *Erm1* had shifted cranially and laterally (Figure 7D,H).

## 4. Discussion

Midfacial clefting is estimated to occur in approximately 5/100,000 live births [35,36]. In human patients, the phenotypic spectrum ranges from a small midline notch of the upper lip to a cleft that separates the central incisors, the medial alveolar ridge, the philtrum, and the nasal septum. The *Unicorn* mouse line is a novel model that can accurately model the human midfacial clefting phenotypes. The *Unicorn* midfacial cleft consists of a bifid nasal septum, and allows for the development of normal bone and cartilage anatomy surrounding the nostrils. These phenotypic features suggest that the *Unicorn* model is both a morphological and genetic tractable model for midfacial clefting. 

The nasal septum is a key growth site responsible for midfacial chondrocranial growth during embryogenesis [37,38,39]. In the case of severe midfacial clefting, such as that observed in the *Unicorn* embryos, we have narrowed the critical period for midfacial morphogenesis to between E10.5 and E11.5. In mandibular development, the condensation of Meckel’s cartilage acts as a scaffold to support the outgrowth of the mandible. The *Unicorn* phenotype suggests that the same principle applies to the nasal septum and the expression of Collagen2-cre can be detected in the nasal region as early as E9.5 [40].

**Figure 8:**
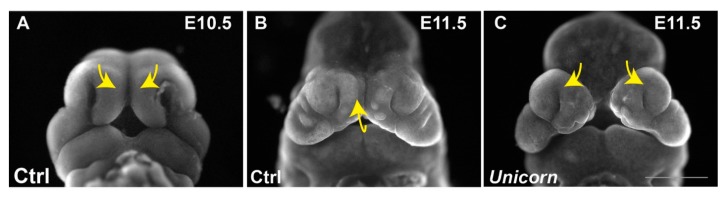
Model for midfacial morphogenesis. Pseudo-SEM images of control and *Unicorn* embryos. (**A**) at E10.5 the MNPs are converging medially followed by (**B**) proximal-distal outgrowth of the merged MNPs. (**C**) The *Unicorn* mutations causes the MNPs to grow laterally rather than medially resulting in the midfacial cleft.

The nasal septum is commonly bifurcated in severe cases of midfacial clefting. At birth, in the mouse and chicken, the nasal midline is supported by a single strut of nasal septum cartilages, while each *Unicorn* nostril is supported by a single strut of nasal septal cartilage. The unique anatomy of the *Unicorn* snout suggests two potential hypotheses regarding early nasal septum development. We hypothesize that the nasal septum may develop as two mesenchymal condensations that must merge in the midline. The alternative is that the bifid nasal septum results from a duplication of a single condensation in the *Unicorn* mutants. Although, our data cannot distinguish between these possibilities, we favor the hypothesis that each medial nasal prominence contributes to half of the nasal septum condensation and subsequent cell movements between E10.5 and E11.5 allow the convergence of the condensation. We also hypothesize that the FEZ directs the morphogenetic movements medially for the MNPs convergence (Figure 8).

Between the bifid nasal septa, we observed the presence of a bone and in histological sections we observed ectopic condensations of bone and cartilage in *Unicorn* mutants (Figure 1, Figure 3 and Figure 4). In normal mice, the superior aspect of the nasal conchae is covered by paired nasal bones. The nasal bones are connected by a suture that is directly above the single nasal septum. We hypothesize that the midline bone in the *Unicorn* mutants is the paired nasal bones that have collapsed into one bone because there was no structural support from the nasal septum to maintain two individual condensations. 

The phenotype of the *Unicorn* mutants suggests that the *Unicorn* Raldh2 mutation is not a full loss of function because the phenotype is discordant from the published *Raldh2*^−/−^ knockout mice. Importantly, the complete knockout of *Raldh2*^−/−^ results in a truncated frontonasal region and is mid-embryonic lethal [17]. Loss of Raldh2 in the frontonasal region results in decreased fibroblast growth factor signaling and Hedgehog signaling in the facial mesenchyme as well as lateral lip clefts, and no midfacial clefts [27]. 

Leo1 is a subunit of the RNA polymerase II complex, and the IMPC phenotyping determined that the mouse homozygotes have a pre-weaning mortality phenotype which precluded any further analysis (http://www.informatics.jax.org/diseasePortal/genoCluster/view/11463). In zebrafish, *Leo1* mutants have decreased craniofacial cartilages especially in the ethmoid plate where it develops a midline cleft [21]. Our mouse *Leo1* expression data suggest that *Leo1* is not expressed ubiquitously, and support the hypothesis that *Leo1* may modulate the transcription of signaling molecules in the telencephalon or MNPs that are required for the induction and patterning of the nasal region.

The paucity of work on normal midfacial morphogenesis and the phenotypic spectrum of human cases further highlights the need to better understand the normal and abnormal midfacial morphogenesis. In conclusion, the *Unicorn* midfacial cleft phenotype has revealed the critical period for the midfacial morphogenesis and is a useful model for beginning to understand the etiology of midfacial clefting. 

## Supplementary Materials

The following are available online at https://www.mdpi.com/2073-4425/11/1/83/s1.
